# Pregnancy-associated plasma protein A – a new indicator of pulmonary vascular remodeling in chronic thromboembolic pulmonary hypertension?

**DOI:** 10.1186/s12931-020-01472-3

**Published:** 2020-08-03

**Authors:** Steffen D. Kriechbaum, Felix Rudolph, Christoph B. Wiedenroth, Lisa Mielzarek, Moritz Haas, Stefan Guth, Christian W. Hamm, Eckhard Mayer, Christoph Liebetrau, Till Keller

**Affiliations:** 1 Department of Cardiology, Campus Kerckhoff of the University of Giessen, Kerckhoff Heart and Thorax Center, Benekestrasse 2-8, 61231 Bad Nauheim, Germany; 2 German Center for Cardiovascular Research (DZHK), Partner Site Rhine-Main, Frankfurt am Main, Germany; 3 Medical Clinic I, Division of Cardiology, Justus Liebig University Giessen, Giessen, Germany; 4 Department of Thoracic Surgery, Campus Kerckhoff of the University of Giessen, Kerckhoff Heart and Thorax Center, Bad Nauheim, 35392 Germany

**Keywords:** PAPP-A, Pregnancy-associated plasma protein A, Pappalysin-1, BPA, PEA, CTEPH, Vascular remodeling

## Abstract

**Background:**

In chronic thromboembolic pulmonary hypertension (CTEPH) impaired pulmonary hemodynamics lead to right heart failure. Natriuretic peptides reflect hemodynamic disease severity. Pregnancy-associated plasma protein-A (PAPP-A) might address another aspect of CTEPH - chronic tissue injury and inflammation. This study assessed dynamics of PAPP-A in CTEPH patients who undergo therapy with pulmonary endarterectomy (PEA) or balloon pulmonary angioplasty (BPA).

**Methods:**

The study included a total of 125 CTEPH patients scheduled for treatment (55 PEA/ 70 BPA) and a control group of 58 patients with pulmonary hypertension other than CTEPH. Biomarker measurement was performed at baseline and follow-up in the CTEPH cohort, prior to each BPA in the BPA cohort and once in the control group.

**Results:**

The median PAPP-A level was slightly higher (*p* = 0.05) in CTEPH patients [13.8 (11.0–18.6) mU/L], than in the control group [12.6 (8.6–16.5) mU/L], without a difference between the BPA and PEA group (*p* = 0.437) and without a correlation to mean pulmonary artery pressure (*p* = 0.188), pulmonary vascular resistance (*p* = 0.893), cardiac index (*p* = 0.821) and right atrial pressure (*p* = 0.596). PEA and BPA therapy decreased the mean pulmonary artery pressure (*p* < 0.001) and pulmonary vascular resistance (*p* < 0.001) and improved the WHO-functional-class (baseline: I:0/II:25/III:80/IV:20 vs. follow-up: I:55/II:58/III:10/IV:2). PAPP-A levels decreased after PEA [13.5 (9.5–17.5) vs. 11.3 (9.8–13.6) mU/L; *p* = 0.003) and BPA treatment [14.3 (11.2–18.9) vs. 11.1 (9.7–13.3) mU/L; *p* < 0.001). The decrease of PAPP-A levels is delayed in comparison to N-terminal pro-B-type natriuretic peptide.

**Conclusion:**

PAPP-A is overexpressed in CTEPH and decrease significantly after surgical or interventional therapy, however without association to hemodynamics. Further investigation is needed to define the underlying mechanism of PAPP-A expression and changes after therapy in CTEPH.

## Introduction

In chronic thromboembolic pulmonary hypertension (CTEPH) insufficient thrombus resolution and vascular remodeling lead to chronic obstructions of the pulmonary arteries [1]. The corresponding impaired pulmonary hemodynamics burden the right heart, cause right heart remodeling and ultimately failure [1]. Pulmonary endarterectomy (PEA), medical treatment targeting pulmonary hypertension (PH) and balloon pulmonary angioplasty (BPA) are specific treatment modalities [1]. Impaired pulmonary hemodynamics in CTEPH correlate with non-invasively measured blood biomarkers such as natriuretic peptides and render such markers as indicators for disease severity and therapy response [2]. Considering the multifaceted pathophysiology of CTEPH, biomarkers not primarily reflecting hemodynamics might further provide information about individual disease mechanisms facilitating treatment decisions. Pregnancy-associated plasma protein-A (PAPP-A), clinically established in the pregnancy first-trimester-screening, was identified as key regulator of insulin-like growth factor (IGF)/IGF-binding-protein pathways via cleaving of IGF-binding-protein [3]. This pathway has been reported in the context of atherosclerosis, coronary artery disease, heart failure and non-cardiac conditions [4]. Its role in PH, especially CTEPH, has not been investigated so far.

The current study aimed to evaluate PAPP-A levels in CTEPH and to explore the potential modification of PAPP-A levels by PEA or BPA treatment.

## Methods

We analyzed 125 consecutive patients with CTEPH and 58 controls (Patients with PH and suspected CTEPH that was excluded after diagnostic workup). Standardized diagnostic and therapeutic work-up of CTEPH patients has been published earlier [2]. The CTEPH group of the study included 55 patients that underwent PEA and 70 in whom BPA was performed. In PEA patients, biospecimen were obtained at baseline and 12 months after surgery (12-MFU), in BPA at baseline, before each staged procedure and 6 months after the final procedure (6-MFU). In control patients, biomaterial was obtained at enrollment. The biomaterial included venous blood samples that were aliquoted and frozen at − 80 °C. The study was approved by the respective local ethics committee and each patient gave written informed consent. PAPP-A was measured in frozen serum samples using an automated immunofluorescence assay on the Kryptor compact plus instrument (PAPP-A Thermo Scientific, BRAHMS GmbH, Henningsdorf, Germany).

Variables are expressed as median (IQR), mean ± SD or number (%) as appropriate. Comparative analyses used the Student t-test, Mann-Whitney-U-test, Wilcoxon signed-rank test, X^2^-test and Fisher-Yates test. Bivariate Pearson correlation assessed associations of PAPP-A with pulmonary hemodynamics and other biomarkers. All *p*-values are seen as descriptive. Statistical analyses were performed with R3.5.1 software package (R Foundation for Statistical Computing, Vienna, Austria).

## Results

The comparative analysis of hemodynamic findings revealed a higher pulmonary artery pressure (meanPAP; 43.1 ± 9.7 vs. 39.9 ± 10.9 mmHg; *p* = 0.041) and pulmonary vascular resistance (PVR; 6.76(5.27–9.61) vs. 4.63(3.15–10.3) WU; *p* = 0.006) in CTEPH patients compared to the PH control group at baseline. A comprehensive illustration of baseline characteristics of the CTEPH cohort and the control group is provided in Table 1.

**Table 1:** Baseline characteristics of the CTEPH cohort and the control group

		CTEPH (total)	CTEPH (BPA)	CTEPH (PEA)	Pulmonary Hypertension other than CTEPH (Controls)	PEA vs. BPA	CTEPH vs. Controls
		***n*** = 125	***n*** = 70	***n*** = 55	***n*** = 58	(***p***-value)	(***p***-value)
	Data availability						
**Demographics**							
Age, y; mean ± SD	183/183	59.3 ± 14.3	60.83 ± 13.5	57.35 ± 15.2	62.28 ± 14.6	0.277	0.14
Female sex; n (%)	183/183	52 (41.6%)	30 (42.86%)	22 (40%)	35 (60.34%)	0.89	0.028
Body mass index, kg/m^2^; mean ± SD	183/183	25.94 ± 4.5	25.09 ± 3.7	27.02 ± 5.1	30.89 ± 7.9	0.046	< 0.001
**History and risk factors**							
Smoker; n (%)	181/183	55 (44.72%)	31 (45.59%)	24 (43.64%)	31 (53.45%)	0.973	0.348
Diabetes mellitus; n (%)	183/183	6 (4.8%)	4 (5.71%)	2 (3.64%)	16 (27.59%)	0.694	< 0.001
Dyslipidemia; n (%)	182/183	23 (18.55%)	17 (24.29%)	6 (11.11%)	14 (24.14%)	0.101	0.499
Arterial hypertension; n (%)	182/183	59 (47.58%)	36 (51.43%)	23 (42.59%)	36 (62.07%)	0.426	0.096
Chronic renal failure; n (%)	183/183	26 (20.8%)	13 (18.57%)	13 (23.64%)	15 (25.86%)	0.638	0.566
Coronary artery disease; n (%)	182/183	20 (16.13%)	14 (20.29%)	6 (10.91%)	12 (20.69%)	0.244	0.586
History of cancer; n (%)	183/183	18 (14.4%)	13 (18.57%)	5 (9.09%)	16 (27.59%)	0.214	0.054
History of acute pulmonary embolism; n (%)	182/183	110 (88.71%)	56 (81.16%)	54 (98.18%)	45 (77.59%)	0.003	0.081
Chronic obstructive pulmonary disease; n (%)	175/183	8 (6.84%)	4 (6.35%)	4 (7.41%)	12 (20.69%)	1	0.014
History of splenectomy; n (%)	183/183	9 (7.2%)	6 (8.57%)	3 (5.45%)	3 (5.17%)	0.73	0.755
Chronic inflammatory disease; n (%)	183/183	3 (2.4%)	1 (1.43%)	2 (3.64%)	6 (10.34%)	0.582	0.030
**Laboratory parameters**							
Ceatinine, μmol/l; mean ± SD	183/183	0.97 ± 0.3	0.95 ± 0.3	1 ± 0.3	0.93 ± 0.4	0.298	0.058
eGFR, ml/min; mean ± SD	183/183	82.5 ± 25.7	83.62 ± 26.6	81.08 ± 24.7	86.63 ± 34.5	0.603	0.558
NT-proBNP, ng/l; median (IQR)	176/183	845 (184.2–1860)	743.7 (197.2–1470)	1094 (149.775–2078.25)	412 (181.8–1454.5)	0.296	0.282
PAPP-A, mU/L	183/183	13.8 (11.0–18.6)	14.5 (11.2–18.9)	13.7 (10.4–17.6)	12.6 (8.6–16.5)	0.437	0.051
**Symptoms and medication**							
Guanylate cyclase stimulator; n (%)	183/183	65 (52%)	49 (70%)	16 (29.09%)	8 (13.79%)	< 0.001	< 0.001
WHO-functional class (I-IV)	183/183	I:0;II:25;III:80;IV:20	I:0;II:11;III:49;IV:10	I:0;II:14;III:31;IV:10	I:0;II:7;III:40;IV:11		
**Examination results**							
LVEF, %; median (IQR)	158/183	55 (55–60)	55 (55–59.25)	55 (55–60)	55 (55–55)	0.416	< 0.001
TAPSE, mm; mean ± SD	153/183	19.08 ± 5.3	18.68 ± 4.8	19.54 ± 5.8	19.5 ± 5.3	0.449	0.788
6-min-walk distance, m; mean ± SD	85/183	405.18 ± 99.1	404.52 ± 91.8	409.44 ± 144.7	329.56 ± 122.3	0.312	0.01
**Hemodynamics**							
RAP, mmHg; median (IQR)	108/183	7 (5–9)	7 (5–9)	7 (5–8)	7.5 (4.5–11.75)	0.977	0.764
MeanPAP, mmHg; mean ± SD	181/183	43.09 ± 9.7	42.44 ± 9.1	43.93 ± 10.6	39.86 ± 10.9	0.384	0.041
PVR, WU (IQR)	172/183	6.76 (5.27–9.61)	6.76 (5.27–8.56)	7.065 (5.3075–11.8075)	4.63 (3.15–10.265)	0.184	0.006
CI, L/min/m^2^; mean ± SD	169/183	2.5 ± 0.6	2.61 ± 0.7	2.33 ± 0.6	2.58 ± 0.8	0.015	0.705
PCWP, mmHg; median (IQR)	179/183	9 (8–12)	9 (8–11)	9 (8–13)	11 (9–13)	0.332	0.004

Values represent N (%) or mean ± SD or median (IQR)

*Abbreviations*: *BPA* Balloon pulmonary angioplasty, *CI* cardiac index, *GFR* glomerular filtration rate, *hs-cTnT* high-sensitivity cardiac troponin T, *LVEF* left ventricular ejection fraction, *NT-proBNP* N-terminal pro-B-type natriuretic peptide, *PAP* pulmonary artery pressure, *PCWP* Pulmonary capillary wedge pressure, *PVR* pulmonary vascular resistance, *RAP* right atrial pressure, *TAPSE* Tricuspid Annular Plane Systolic Excursion

PAPP-A levels at baseline were comparable in CTEPH patients [13.8(IQR 11.0–18.6) mU/L] and PH controls [12.6(IQR 8.6–16.5) mU/L] (*p* = 0.051). No relevant correlation between PAPP-A and hemodynamic parameters such as meanPAP (*r* = 0.120; *p* = 0.188), pulmonary vascular resistance (PVR) (*r* = 0.013; *p* = 0.893) or NT-proBNP (*r* = 0.128; *p* = 0.169) was observed in CTEPH patients. Nevertheless, PAPP-A correlated with C-reactive protein (*r* = 0.259; *p* = 0.004).

Surgical and interventional treatment led to an improvement of pulmonary hemodynamics and a decrease of natriuretic peptides, which is illustrated in Table 2.

**Table 2:** Comparison of hemodynamic findings and NT-proBNP levels between baseline and follow-up in CTEPH patients

	Baseline mean ± SD or median (IQR)	Follow-up mean ± SD or median (IQR)	***p***-value
**PEA**			
MeanPAP; mmHg	43.9 ± 10.6	22.3 ± 7.5	p < 0.001
PVR; WU	7.1 (5.3–11.8)	2.5 (1.8–3.5)	p < 0.001
NT-proBNP; ng/L	1094 (150–2078)	192 (102–382)	p < 0.001
**BPA**			
MeanPAP; mmHg	42.4 ± 9.1	31.7 ± 9.6	p < 0.001
PVR; WU	7.1 (5.3–11.8)	3.9 (3.1–5.3)	p < 0.001
NT-proBNP; ng/L	744 (197–1470)	121 (70–238)	p < 0.001

Values represent as mean ± SD or median (IQR)

*Abbreviations*: *BPA* Balloon pulmonary angioplasty, *NT-proBNP* N-terminal pro-B-type natriuretic peptide, *meanPAP* mean pulmonary artery pressure, *PEA* pulmonary endarterectomy, *PVR* pulmonary vascular resistance

**Fig. 1:**
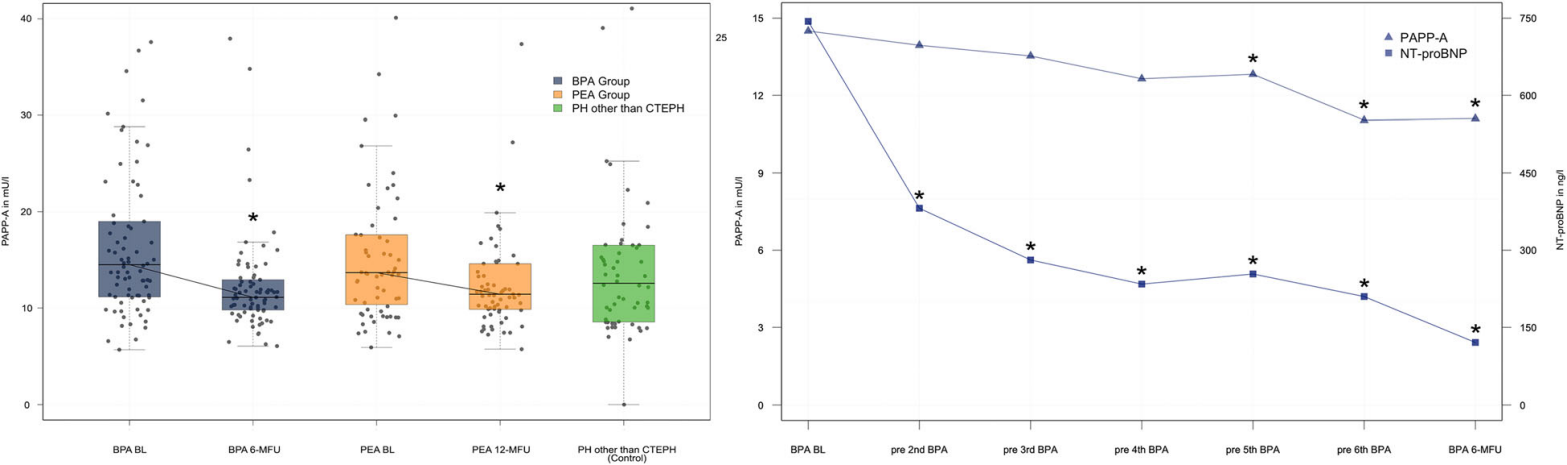
Impact of treatment on pregnancy-associated plasma protein A (PAPP-A) levels in patients with chronic thromboembolic pulmonary hypertension (CTEPH)*.* The left panel shows PAPPA-A levels in CTEPH patients before undergoing treatment with pulmonary endarterectomy (PEA BL) or balloon pulmonary angioplasty (BPA BL) and 12 months after PEA (PEA 12-MFU) respectively 6 months after the final BPA (BPA 6-MFU) procedure compared to controls of patients with pulmonary hypertension in whom CTEPH was excluded (PH Controls). The right panel shows the time dependent effect of the staged BPA procedure on PAPP-A levels. For comparison, data on NT-proBNP as a biomarker reflecting hemodynamics is provided. * Indicates *p*-value < 0.05 comparing difference in PAPP-A level at procedure compared to the baseline level

The PAPP-A levels did not differ between the PEA and BPA treatment group (13.7 (10.4–17.6) vs. 14.5 (11.2–18.9) mU/L; *p* = 0.437) at baseline (Fig. 1*, left panel*). PAPP-A levels decreased significantly after treatment from 13.7 (10.4–17.6) to 11.4 (9.9–14.6) mU/L (*p* = 0.003) after PEA and 14.5 (11.2–18.9) to 11.1 (9.8–12.9) mU/L (*p* < 0.001) after BPA therapy (Fig. 1*, left panel*).

BPA is a staged procedure (median 6 procedures/patient) with only a limited number of pulmonary segments treated per session. PAPP-A levels decreased continuously reaching a significant change after 4 procedures in contrast to the hemodynamic marker NT-proBNP as a comparator that was significantly lowered already after the first procedure (Fig. 1*, right panel*).

## Discussion

Key findings of this study are: (1) PAPP-A levels might be associated with CTEPH and decrease after interventional or surgical treatment. (2) The PAPP-A treatment response shows a slow and continuous lowering in marker levels in contrast to the rapid improvement in hemodynamics reflected by biomarkers such as NT-proBNP.

The PAPP-A levels in CTEPH patients, which are modifiable by treatment. Seem not to be mediated by hemodynamics and their improvement after treatment raising the question about the origin and role of PAPP-A in CTEPH.

Acute pulmonary embolism impairs pulmonary vascular homeostasis. The mechanisms leading to development of CTEPH in a subset of PE patients are not fully understood. Endothelial damage, dysfunction and inflammation are known to be involved in vascular remodeling. The IGF-I/IGF-receptor signaling promotes inflammation, anti-apoptosis and proliferation in various cell types such as endothelial and smooth muscle cells [5, 6]. Yang et al. reported a key role of the IGF-I/IGF-receptor signaling in neonatal PH, revealing an upregulation of IGF-I expression in pulmonary endothelial and smooth muscle cells under experimental hypoxia [7, 8]. Further, Harrington et al. identified PAPP-A as a promotor of atherosclerotic plaque progression and plaque vulnerability. This processes seemed to be driven by PAPP-A-mediated proinflammatory effects of macrophage cytokines and a consecutive upregulation of the IGF-I/IGF-receptor axis [9].

One might hypothesize, that an overexpression of PAPP-A might thus reflect chronic vascular remodeling in CTEPH. The potential use as a biomarker indicating disease mechanisms other than hemodynamics is further supported by the availability of robust automated measurement technology due to the routine use in the context of pregnancy. Further, as the IGF pathway plays a relevant role in certain cancer entities, PAPP-A has already been discussed as treatment target that led to e.g. development of monoclonal PAPP-A antibodies [10].

The present clinical study is based on relatively small cohort and therefore the results only allow to hypothesize about the potential role of PAPP-A. However, it is the first analysis showing an association of PAPP-A with CTEPH. Especially the modification of PAPP-A levels by treatment not primary mediated via hemodynamic improvement should stimulate further investigations to confirm the present results from a small cohort and further analyze the specific role of PAPP-A in the pathophysiology of CTEPH and a potential clinical use as biomarker or even treatment target.

## Data Availability

The data underlying this article cannot be shared publicly due to the privacy of individuals that participated in the study. Sharing the underlying data is not in line with the written informed consent of the patients in this study. The data will be shared on reasonable request to the corresponding author.

## Abbreviations

12-MFU: Follow-Up twelve month after PEA
6-MFU: Follow-Up six month after last BPA
BPA: Balloon pulmonary angioplasty
CTEPH: Chronic thromboembolic pulmonary hypertension
eGFR: estimated glomerular filtration rate
IGF(−I): Insulin-like growth factor (− 1)
IQR: Interquartile range
meanPAP: Mean pulmonary artery pressure
NT-proBNP: N-terminal pro brain natriuretic peptide
PAPP-A: Pregnancy-associated plasma protein A or Pappalysin-1
PCWP: Pulmonary capillary wedge pressure
PEA: Pulmonary endarterectomy
PH: Pulmonary hypertension
PVR: Pulmonary vascular resistance
SD: Standard deviation
WU: Wood units
